# Biomineralization of Plastic Waste to Improve the Strength of Plastic-Reinforced Cement Mortar

**DOI:** 10.3390/ma14081949

**Published:** 2021-04-13

**Authors:** Seth Kane, Abby Thane, Michael Espinal, Kendra Lunday, Hakan Armağan, Adrienne Phillips, Chelsea Heveran, Cecily Ryan

**Affiliations:** 1 Mechanical and Industrial Engineering Department, Montana State University, Bozeman, MT 59717, USA; michael.espinal@student.montana.edu (M.E.); chelsea.heveran@montana.edu (C.H.); cecily.ryan@montana.edu (C.R.); 2 Center for Biofilm Engineering, Montana State University, Bozeman, MT 59717, USA; abbyathane@gmail.com (A.T.); adrienne.phillips@montana.edu (A.P.); 3 Capital High School, Helena, MT 59601, USA; klunday@helenaschools.org; 4 Omaha Burke High School, Omaha, NE 68154, USA; sezai.armagan@ops.org; 5 Civil Engineering Department, Montana State University, Bozeman, MT 59717, USA

**Keywords:** plastic waste, cement, concrete, microbial-induced calcium carbonate precipitation, valorization, plastic-reinforced concrete, plastic recycling

## Abstract

The development of methods to reuse large volumes of plastic waste is essential to curb the environmental impact of plastic pollution. Plastic-reinforced cementitious materials (PRCs), such as plastic-reinforced mortar (PRM), may be potential avenues to productively use large quantities of low-value plastic waste. However, poor bonding between the plastic and cement matrix reduces the strength of PRCs, limiting its viable applications. In this study, calcium carbonate biomineralization techniques were applied to coat plastic waste and improved the compressive strength of PRM. Two biomineralization treatments were examined: enzymatically induced calcium carbonate precipitation (EICP) and microbially induced calcium carbonate precipitation (MICP). MICP treatment of polyethylene terephthalate (PET) resulted in PRMs with compressive strengths similar to that of plastic-free mortar and higher than the compressive strengths of PRMs with untreated or EICP-treated PET. Based on the results of this study, MICP was used to treat hard-to-recycle types 3–7 plastic waste. No plastics investigated in this study inhibited the MICP process. PRM samples with 5% MICP-treated polyvinyl chloride (PVC) and mixed type 3–7 plastic had compressive strengths similar to plastic-free mortar. These results indicate that MICP treatment can improve PRM strength and that MICP-treated PRM shows promise as a method to reuse plastic waste.

## 1. Introduction

Plastic is one of the world’s largest and fastest-growing waste streams, with 368 million tons of plastic waste generated in 2019 [1]. Plastic recycling rates remain low, with only 9% of plastics recycled, despite strong public interest [2]. The amount of plastic recycled is economically limited by the low cost of virgin plastic and the high costs associated with recycling processes such as transportation, sorting, cleaning, and extrusion. This limitation leads to low-value type 3–7 plastics typically being routed to the landfill or being improperly managed, thereby contaminating the environment. New approaches are urgently needed to reduce the volume of plastic waste sent to landfills and entering the environment.

The addition of waste materials to concrete has the potential to reuse large volumes of diverse waste streams including glass, plastic, and industrial waste [3,4,5,6]. The addition of waste to concrete provides the dual benefits of redirecting waste away from the landfill and reducing greenhouse gas emissions associated with cement production. In particular, plastic-reinforced cementitious materials (PRCs), such as plastic-reinforced mortar (PRM), may allow for the repurposing of mixed-type plastic waste of varying geometries, eliminating the costly sorting process required for conventional plastic recycling [3,7,8,9,10,11,12,13,14,15]. The use of PRCs is currently limited by the low strength of these composites relative to conventional concrete [3,10]. This decrease in strength further declines with increasing volumes of plastic addition, constraining the amount of plastic waste that can be added to PRC. Three mechanisms have been proposed to explain this decrease in strength: (1) stress concentrations due to the low modulus of plastic relative to the cement matrix, (2) increased porosity with plastic addition, and (3) poor bonding between the plastic and cement matrix [3]. Several studies have attempted to improve the strength of PRC by improving the interfacial strength via pretreatment of the plastic [8,16,17,18]. One particularly promising treatment is the deposition of calcium carbonate (CaCO_3_) onto the surface of the plastic. Both microbial and abiotic methods have been used to deposit CaCO_3_. Both methods increase the interfacial strength between polypropylene and the cement matrix and bending strength and locally increase cement hydration [17,18]. However, critical gaps remain in further evaluating the potential of CaCO_3_ treatment to improve PRM strength. Notably, it is unknown how the method of CaCO_3_ deposition used affects PRC strength, including the potential impacts of biofilms formed during microbial CaCO_3_ treatment. Past studies have also been limited by examining only CaCO_3_ treatment of polypropylene plastic. It is necessary to examine whether CaCO_3_ pretreatment of other plastic types with limited markets for recycling would also benefit PRM strength.

Biomineralization is one method to deposit CaCO_3_ onto plastic. During CaCO_3_ biomineralization, the urease enzyme catalyzes the hydrolysis of urea to precipitate CaCO_3_. In the microbially induced calcium carbonate precipitation (MICP) process, the urease enzyme is produced by microorganisms, such as the soil bacterium *Sporosarcina pasteurii* (*S. pasteurii*) [19,20,21]. If urease enzymes are instead sourced from *Canavalia ensiformis* (i.e., jack bean), the process is termed enzymatically induced calcium carbonate precipitation (EICP) [22]. Both techniques have been applied to repair cracks in concrete and to seal leaky oil wells [19,23,24,25,26,27]. Either EICP or MICP could potentially be leveraged to control the deposition of CaCO_3_ onto waste plastic, and both may be easier to scale at low cost than past techniques to deposit CaCO_3_ onto plastic [17]. Prior to this study, it was not yet known if EICP- and MICP-precipitated CaCO_3_ may differently affect the strength of PRM.

In this study, we examine the impact of CaCO_3_ biomineralization of plastic on PRM. We first compared EICP and MICP biomineralization techniques to determine the impact of the biofilm in MICP treatment on CaCO_3_ deposition onto plastic and the strength of the resulting PRM. To this end, mortar samples were reinforced with 1% and 5% of untreated polyethylene terephthalate (PET), MICP-treated PET, and EICP-treated PET and the compressive strength of the resulting PRM was evaluated. We then explored the feasibility of creating a CaCO_3_ coating on the surfaces of type 3–7 and mixed type plastic, which have limited recycling markets. These plastics were mineralized with MICP to determine if any plastic types are inhibitory to the mineralization process. MICP-treated plastics were then added to cement mortar at 5% plastic, and the compressive strength of the resulting PRM was compared with untreated PRM and non-reinforced (plastic-free) mortar. We hypothesized that MICP treatment of waste plastic would improve the compressive strength of PRM relative to PRM prepared with unmineralized plastics. This study further develops CaCO_3_ biomineralization as a method to improve the strength of PRM by identifying the impact of biofilm formation on CaCO_3_ deposition. By examining the application of MICP treatment to more plastic types than have been examined in past studies, this study establishes MICP as a potential treatment for other hard-to-recycle plastic types.

## 2. Materials and Methods

### 2.1. Materials

#### 2.1.1. Plastic

Polyethylene terephthalate (PET) plastic additive was prepared by grinding PET clamshell food containers in a commercial laboratory blender (Model # 34BL97, Waring, Stamford, CT, USA) for two minutes at medium speed. The ground particles were passed through a 2 mm sieve followed by a 0.85 mm sieve to achieve a size fraction between these two values. This ensured that particle sizes were relatively uniform and did not exceed one-third of the minimum dimension of the mold that would be used to make future mortar specimens [28].

For batch-test comparisons to test for potential inhibition to the biomineralization process by uncommonly recycled plastic, polyvinyl chloride (PVC, type 3), low-density polyethylene (LDPE, type 4), polypropylene (PP, type 5), polystyrene (PS, type 6), and acrylonitrile butadiene styrene (ABS, type 7) plastic flakes were provided by Northwest Polymers (Molalla, OR, USA).

These flakes differed in geometry by plastic type. To directly compare the amount and morphology of biomineral formed on each plastic type during mineralization, the flakes were molded into disks of uniform size and thickness. Constant volumes of flakes were hot-pressed between two films of mylar into thin sheets on a hot press (Model # LCB1015-3, Auplex, Fuzhou, Fujian, China) at the temperatures shown in Table S1. Disk samples of 0.6 cm in diameter were cut from the thin sheets using a standard paper hole punch.

The geometries of ground, recycled plastic flakes for type 3–7 plastics varied by plastic type. To eliminate this uncontrolled variable of flake geometry, chopped 1.75 mm plastic filament was used to examine the effect of mineralization on PRM reinforced with type 3–7 plastics. Plastic filament was purchased from https://Filaments.ca (Mississauga, ON, Canada, accessed on 4 August 2020) Canada) for the PVC (Filamentum Vinyl 303 PVC—Black), LDPE (Filaments.ca LLDPE Filament—Natural), PP (Centaur Polypropylene Filament—White), PS (NefilaTek 100% Recycled HIPS Filament—Black), and ABS (NefilaTek 100% Recycled ABS Filament—Black) plastic types. The filament was cut into approximately 0.8 cm lengths with a goal upper limit of 1.6 cm using a paper cutter fitted with a 3D-printed cutting jig. This length was chosen to meet the fiber length reinforcement requirements specified for beam samples in ASTM International C1609 [28]. Before use, all plastics were disinfected by submerging in 70% ethanol for ten minutes and by rinsing three times with sterile deionized (DI) water. The density, tensile strength, and elastic modulus of each polymer fiber is described in Table S1.

For each type of plastic, random samples of 50 fibers were measured with calipers to estimate their overall length distribution. A normal Gaussian fit of the distribution of fiber lengths for each plastic type is shown in Figure S2. All mean lengths were within one standard deviation of the 8 mm goal length.

#### 2.1.2. Mineralization Solutions

All chemicals were purchased through Fisher Scientific unless otherwise noted. The following solutions were used to grow *S. pasteurii*: brain heart infusion (BHI) media composed of 37 g/L brain heart infusion broth spiked with 20 g/L urea and calcium mineralizing media containing 3 g/L nutrient broth, 10 g/L ammonium chloride, 20 g/L urea (CMM−), adjusted to pH 6.15 and spiked with 49 g/L calcium chloride dihydrate (CMM+). All solutions were prepared with DI water and filtered with sterile syringe filters with 0.2 μm cellulose acetate membrane (VWR part # 28145-475, Radnor, PA, USA) before use.

For EICP tests, a similar solution composed of urea (20 g/L) and calcium chloride dihydrate (49 g/L) (U + C) in DI water that did not include nutrients necessary for microbial growth was used. This solution was prepared immediately before use and was not filter sterilized.

### 2.2. Batch Testing for Microbial Growth and Plastic Biomineralization

Batch tests were completed for the PET flakes and type 3–7 disks (1) to determine if *S. pasteurii* cells can attach to the surface of each plastic during MICP, (2) to evaluate if the addition of plastics inhibits biomineralization processes, and (3) to examine the physical characteristics and amount of precipitates on each type of plastic. No inhibition batch tests were performed on the EICP process as it was observed that the MICP process was not inhibited under the conditions and time scale tested.

#### 2.2.1. MICP Biomineralization of PET

The following conditions were tested in triplicate using PET flakes: (1) *S. pasteurii* with PET (test condition), (2) *S. pasteurii* without PET (positive control), an (3) PET without *S. pasteurii.* (negative control) (Table 1). An *S. pasteurii* (ATCC 11859) starter culture was prepared in brain heart infusion (BHI) and precipitation was promoted using calcium mineralizing medium (CMM) as described in past studies [29,30].

PET plastic flakes (1 g) and either 1 mL of *S. pasteurii culture* or no bacteria (negative control) were added to Erlenmeyer flasks filled with 100 mL BHI. The flasks were incubated on a shaking table at room temperature (Innova 2400 Platform Shaker, New Brunswick Scientific, Enfield, CN, USA). The samples were collected for optical density at 600 nm (OD${}_{600}$) measured in triplicate on 200 μL in a 96-well plate by a Biotek spectrophotometer at 0, 1, 2, 4, 8, and 24 h, and pH and urea concentration were measured at 0, 8, and 24 h (Fisher Accumet probe, AR20 Accumet pH and conductivity meter, Fisher Scientific, Waltham, MA, USA). The samples were filtered (VWR sterile syringe filters with 0.2 μm cellulose acetate membrane) and stored at 4 °C before assessment of the urea concentration using a modified Jung colorimetric assay [31,32].

#### 2.2.2. Types 3–7 Biomineralization Comparisons

More extensive batch tests were completed for plastic types 3–7. The tests were conducted in two phases: (1) a 24 h attachment phase to evaluate biofilm growth on plastics and (2) a 24 h mineralization phase to assess biomineral formation. Three conditions (Table 1) were tested in triplicate. In phase 1, twenty plastic discs were added to each of the test and negative control flasks filled with 100 mL CMM for each of type 3–7 plastics. The flasks were incubated and sampled at 0, 4, 8, and 24 h for pH, OD${}_{600}$, and urea concentration as described above.

After the 24 h biofilm growth phase, 19 disks from each flask were transferred to 250 mL flasks with 100 mL CMM+ for the mineralization phase. The flasks were shaken for 24 h at room temperature. At 0 and 24 h, the samples were collected for pH and urea concentration analysis as described above.

#### 2.2.3. Calcium Digests

A calcium digest was performed to estimate the mass of calcium carbonate formed on the plastic. For PET plastic mineralized with MICP, 10 g PET flakes were added to 100 mL BHI inoculated with a 1 mL *S. pasteurii* cryo-stock and shaken at 150 rpm and 30 °C for 24 h. The plastic was then transferred to 100 mL CMM+ and shaken for an additional 24 h. For PET plastic mineralized with EICP, 5 g PET flakes were added to 100 mL U + C with 5 g/L jack bean meal (jack bean fine powder, Sigma-Aldrich product #J0125, St Louis, MO, USA) as the urease source and shaken at room temperature for 24 h. After mineralization, the plastic was dried at room temperature for 24 h. Three samples of plastic weighing 1 g each were acid digested using the procedure described above to assess the mass of calcium.

For plastic types 3–7, three disks from each flask were weighed and added to a 15 mL centrifuge tube containing 5 mL 10% trace metal grade nitric acid (Fisher Scientific, A509-P212). The tubes were vortexed and left for 24 h at room temperature. The supernate was then removed from each tube, serially diluted, and analyzed for calcium concentration using a colorimetric calcium assay [33]. The disks were dried at room temperature for 24 h and weighed a second time. The weight difference before and after digestion was compared to the mass of calcium carbonate calculated from the concentration found in the supernate.

#### 2.2.4. X-ray Diffraction Spectroscopy

Untreated PET, EICP-PET, and MICP-PET samples were analyzed with a SCINTAG X1 X-ray Powder Diffraction Spectrometer (XRD) to identify calcium carbonate polymorphs. The samples were held on a glass slide with Vaseline and analyzed from 2-θ of 3–75°.

### 2.3. Preparation and Testing of Plastic-Reinforced Mortar Specimens

Cement mortar cylinder specimens were prepared for the test conditions described in Table 2. The impacts of EICP- and MICP-treated PET were assessed at 1 and 5 wt.%; for plastic types 3–7 individually and mixed, and the impact of MICP-treated plastics were only evaluated at 5 wt.%.

#### 2.3.1. Mineralization of Plastic

PET flakes were mineralized via EICP as described in Section 2.2.1.

PET flakes were treated with MICP by adding flakes to flasks containing BHI inoculated with *S. pasteurii*. After shaking at 150 rpm and 30 °C for 24 h, the fluid was strained out of the BHI culture and drained, and CMM+ was added for an additional 24 h treatment at room temperature. The plastic was then strained out of the solution and dried at room temperature.

Type 3–7 plastics (treated individually and as an equal mass mixture) were treated with MICP by placing 121 g of plastic into a mesh bag that was submerged in 700 mL of CMM+ in a 1 L beaker on a stir plate (MIRAK, Barnstead Thermolyne), mixing at 140 rpm (Figure S1). The beakers were inoculated with 14 mL of an *S. pasteurii* culture. The beakers were covered loosely with aluminum foil and incubated for 48 h at room temperature. After mineralization, the fluid was drained and the plastic fibers were dried at room temperature for 24 h.

#### 2.3.2. Cement Mortar Production

Cement mortar cylinders are prepared for the experimental conditions shown in Table 2. Mortar specimens were prepared following a procedure based on ASTM International C109 [34]. Each batch was mixed with 2500 g of ordinary Portland cement (Quikrete, commercial grade) with 0 wt.%, 1 wt.%, or 5 wt.% of cement replaced with the equivalent weight of plastic. To control for the amount of plastic between untreated and mineralized plastic, the plastic was weighed pre-mineralization. A cement:sand ratio of 0.8 and a water:cement ratio of 0.46 were used for all batches. Cement mortar was mixed following the procedure described in ASTM International C305 [35].

Cylinder molds (2 in D × 4 in H (5.08 cm × 10.1 cm); Bio-cylinder, Deslauriers Inc., La Grange Park, IL, USA) were sprayed with a thin coat of vegetable oil before the addition of cement mortar. The mortar was added to the molds following the procedure described ASTM C192 [36]. After molding, the specimens were stored in a concrete curing room at 100% relative humidity until testing. Specimens were demolded 24 h after mixing and returned to the cement curing room until testing.

#### 2.3.3. Cement Mortar Compressive Testing

Strength testing was completed for cylinder specimens with an MTS Criterion Model 64 load frame on days 14 and 28 of curing. Due to instrument maintenance issues, PET specimens and their controls were tested on day 35 rather than day 28. Specimen strengths are not expected to change after 28 days of curing. Neoprene caps were placed over the end of the cylinder specimens, and the specimens were compressed between the lower crosshead and the test table. Compression was performed at a constant rate of 0.127 mm/s (0.005 in/s) until failure.

### 2.4. Microscopy

Mineralized plastic samples and PRM samples were examined on a Field Emission Scanning Electron Microscope (FESEM). The samples were placed on carbon sticky dots, sputter-coated with gold on an Emitech K-875X Sputter Coater, and examined on a Zeiss Supra 55VP FESEM at 1 kV and a working distance of 3.9–5.3 mm with an SE2 detector.

Confocal images of *S. pasteurii* attachment to PET were collected using a Leica CS upright confocal microscope (TCS SP5 IIDM6000, Leica Microsystems, Buffalo Grove, IL, USA), as described in Section S1.1 of the Supplementary Materials.

### 2.5. Characterization of Cement Hydration and Structure

Cement paste samples were prepared to determine the effect of MICP-produced biomineral on the degree of hydration and cement crystalline structure. Mixed type 3–7 plastics were mineralized following the submersion method in Section 2.3.1. To obtain a macroscopically homogeneous cement paste sample, the biomineral was scraped off of the plastic and ground to a powder. The cement paste samples were prepared with a 0.46 water:cement ratio and no sand, with 0%, 1%, and 5% of the cement replaced with biomineral. Sample mixture followed the procedure described in Section 2.3.2. Samples were molded in weigh boats (Thermo Scientific, part #08-732-112) and cured as in Section 2.3.2. After 1, 7, 14, and 28 days of curing, a sample of each mineral amount was removed from the curing room, allowed to dry, and ground into a powder with a mortar and pestle for use in the thermogravimetric analysis (TGA) and XRD measurements.

TGA was performed on a TA Instruments Q5000-IR TGA (TA Instruments Inc., New Castle, DE, USA) to determine the degree of hydration of the cement paste samples. Triplicate samples of 40–50 mg of cement paste powder were placed in TA Instruments high-temperature platinum TGA pans for each of the 0%, 1%, and 5% biomineral samples. The samples were heated from 30 °C to 1000 °C at 10 °C/min. Weight loss data were analyzed to determine the degree of hydration following the method described by Monteagudo et al., with modification of decarbonation beginning at 390 °C rather than 400 °C to include the full TGA derivative peak observed at that temperature [37,38]. A value of 24% was used to correspond to complete hydration [38].

XRD measurements were taken of the cement paste samples as in Section 2.2.4. Jade (Materials Data Inc., Livermore, CA, USA). was used to identify the peaks associated with the phases present within both the biomineral and hydrated cement phases. Whole pattern matching was used to determine the relative semi-quantitative phase composition of the hydrated cement with and without biomineralized plastic [39,40,41,42,43].

### 2.6. Data Analysis

All statistical analyses were performed in Minitab (vers. 19.2020.1, Minitab LLC, State College, PA, USA). Critical alpha was set a priori to 0.05 for all analyses. The effect of the addition of untreated PET on mortar compressive strength was evaluated using one-way ANOVA. Two-factor ANOVA evaluated the effects of the amount of PET and type of mineralization treatment (MICP and EICP) on the dependent variables of compressive strength and modulus. Additional models compared the effects of plastic type and mineralization type on PRM strength and modulus. For all models, the residuals satisfied the assumptions of normality and homoscedasticity. Follow-up testing was performed using the Bonferonni method, where critical alpha was divided by the number of comparisons to control family-wise error.

## 3. Results and Discussion

### 3.1. Comparison of EICP and MICP Treatment of PET

**Figure 1:**
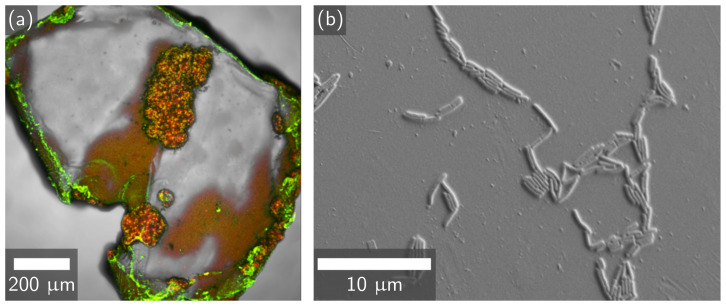
(**a**) Confocal image of *S. pasteurii* attachment and CaCO_3_ precipitation of a MICP-treated PET flake at 10× magnification. The *S. pasteurii* biofilm is stained green, biomineral deposits are stained red, and yellow represents regions where both the biofilm and biomineral are present. (**b**) FESEM image of *S. pasteurii* bacteria attachment to a PET flake at 4700× magnification.

In the first study comparing EICP and MICP treatment, PET did not impair the growth of *S. pasteurii* in a flask culture. This was shown by OD${}_{600}$ and pH measurements that matched those of the no-PET control over a 24 h growth study (Figure S3). Urea concentration decreased and pH increased at similar rates over time both with and without PET (Figure S3), indicating that the reaction was not impaired by plastic. Attachment of *S. pasteurii* to the PET flakes was observed via both confocal microscopy and FESEM (Figure 1). This demonstrates that *S. pasteurii* was successful in forming biofilms on the PET flakes under the conditions tested.

**Figure 2:**
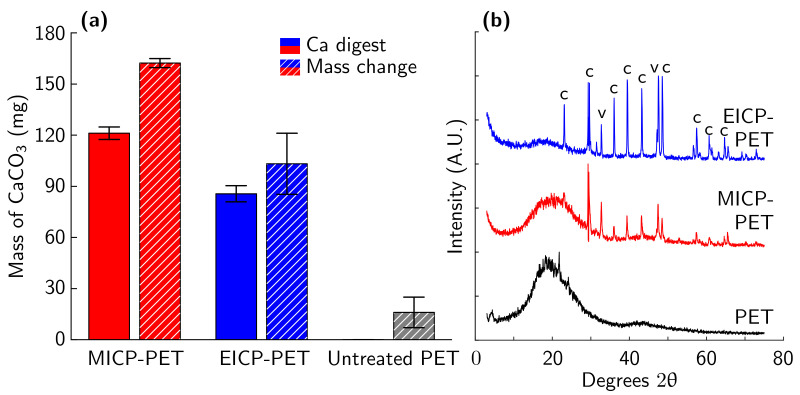
(**a**) Calcium assay and mass change means for MICP-treated PET flakes, enzymatically induced calcium carbonate precipitation (EICP)-treated PET flakes, and a control sample of untreated PET flakes. The error bars indicate one standard deviation. (**b**) XRD spectra for PET flakes (black), EICP-treated PET flakes (blue), and MICP-treated PET flakes (red). Peaks labeled c represent the calcite phase of CaCO_3_, while peaks labeled v represent the vaterite phase. No aragonite phases or other minerals were observed.

**Figure 3:**
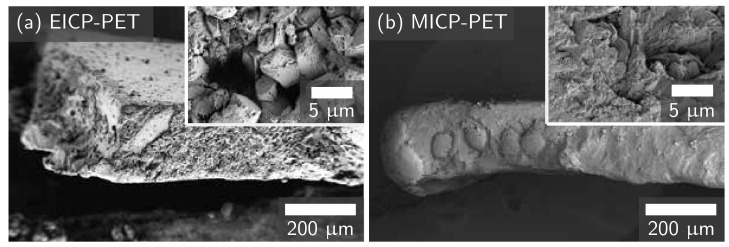
FESEM images of EICP-treated PET (**a**) and MICP-treated PET (**b**) at approximately 300× magnification. Inset images show details of the CaCO_3_ texture at approximately 10k× magnification. An image of an untreated control sample is shown in Figure S4.

Both MICP and EICP formed a CaCO_3_ coating on PET flakes. MICP treatment deposited more CaCO_3_ on PET than EICP treatment as measured by both calcium assay and mass change (Figure 2a). XRD measurements showed similar spectra for both EICP and MICP. Both mineralization methods were observed to have calcite and vaterite phases of CaCO_3_ (Figure 2b). The observed broad amorphous bands are attributed to the PET. No other phases of CaCO_3_ or other minerals were identified. This finding is consistent with past work that found that EICP can produce the calcite phase of CaCO_3_ [44,45] while MICP may precipitate vaterite as a transient, meta-stable, lower-modulus phase that eventually transforms to calcite [44,46,47]. FESEM imaging of the biomineral shows evidence that EICP produced faceted polycrystals with a length of approximately 1–5 μm (Figure 3a). In contrast, images of MICP-treated PET showed evidence of predominantly large, rounded structures composed of very small polycrystals (<1 μm facets) (Figure 3b).

**Figure 4:**
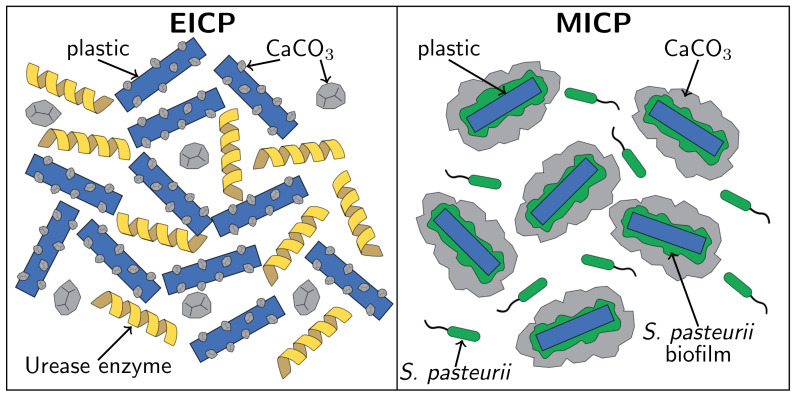
Proposed mechanism for the differences between EICP and MICP biomineralization of plastic. The formation of a biofilm on the plastic in MICP localizes CaCO_3_ precipitation to the surface of the plastic, while EICP biomineralization is more evenly distributed throughout the solution.

The differences between EICP and MICP biomineral morphology may depend on the influence of bacteria. Smaller CaCO_3_ crystals are produced at higher saturation states, and microorganisms in MICP would locally increase saturation state [46]. During EICP, urease enzyme from jack bean would be expected to affect the solution more uniformly [46]. Furthermore, these bacteria can produce extra polymeric substances that may stabilize forming surfaces [19,23]. This proposed mechanism is shown in Figure 4. MICP’s greater deposition of CaCO_3_ on PET could result from the attachment of bacteria to PET (Figure 1 and Figure 4) and then nucleation of CaCO_3_ on bacterial cell walls [20,21,48]. In contrast, during EICP, CaCO_3_ forms without these benefits from microbial cells.

**Figure 5:**
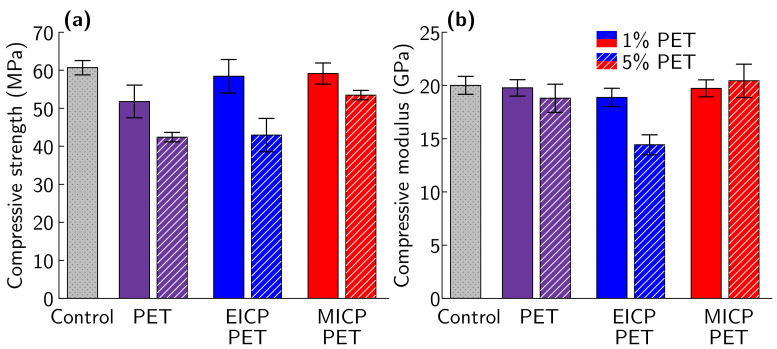
Comparison of (**a**) compressive strength and (**b**) compressive modulus for non-reinforced mortar and mortar reinforced with 1% and 5% untreated PET, 1% and 5% EICP-treated PET, and 1% and 5% MICP-treated PET after 35 days of curing. Values for 14 and 35 day compressive strength and modulus are listed in Table S2. Representative stress-strain curves for these sample types are shown in Figure S6.

After 35 days of curing, compressive strength decreased for PRM with untreated PET (*p* <0.001, Figure 5a). PRM samples with 5% PET showed a larger decrease in strength than PRM samples with 1% PET (Table 3, *p* <0.01). This is consistent with prior reports of losses in strength for similar additions of untreated PET to cement mortar [3]. Two-way ANOVA testing showed a significant interaction (*p* <0.05) between the effect of the amount of PET replacement and mineralization technique (EICP or MICP) on PRM compressive strength. Post hoc testing found that PRM samples with 1% EICP-treated PET and 1% MICP-treated PET both show similar compressive strengths to non-reinforced mortar samples (Table 3, *p* >0.05 for each). In contrast, PRM samples with untreated PET were found to have significantly lower compressive strength than non-reinforced mortar samples (Table 3, *p* <0.05). At the 5% PET level, EICP-treated PET and untreated PET reinforced mortar show similar compressive strengths (*p* >0.05) while PRM samples with MICP-treated PET show significantly higher strength than either EICP-treated PET or untreated PET (*p* <0.05 for both). No treatments or amounts of PET added to PRM samples had a significant effect on compressive modulus (*p* >0.05, Figure 5b). Similar trends are seen for compressive strength and modulus measurements taken after 14 days (Table S2).

**Figure 6:**
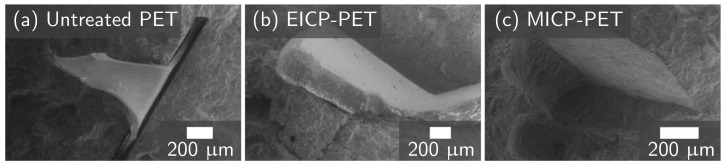
FESEM images of the interface between PET flakes and the cement mortar for (**a**) untreated PET, (**b**) EICP-treated PET, and (**c**) MICP-treated PET.

One possible explanation for the strength differences could be that the untreated PET was not well bonded to the cement matrix. FESEM imaging of the failure interface of PRM with untreated PET provides evidence to support this explanation, with a gap visible at the interface for the assessed sample (Figure 6a). In contrast, images of both EICP-treated PET (Figure 6b) and MICP-treated PET (Figure 6c) showed a continuous interface between the mineralized plastic and the surrounding cement matrix. This improved contact along with the observed increase in compressive strength supports the hypothesis that biomineralization of plastic waste could improve the interfacial strength between plastic waste and cement. However, additional work directly measuring the effect of mineralization on interfacial strength is needed to further test this hypothesis.

These results show that, at 1% plastic replacement, both EICP and MICP mineralization of PET results in comparable PRM compressive strengths that are higher than those of mortar reinforced with untreated PET. Because MICP requires additional infrastructure to culture cells, this indicates that EICP may be an appropriate biomineralization choice for low-volume PET applications. An important result is that mortar reinforced with 5% treated PET shows significant improvements in strength over both EICP-treated PET and untreated PET, with compressive strengths similar to 1% untreated PET-reinforced mortar. This result shows that MICP treatment allows for the use of higher volumes of plastic, with less loss in strength than would be expected in untreated plastic. In applications where density is of concern, 5% MICP-treated PET mortar shows even more promise due to the decrease in density with the addition of higher quantities of PET [3].

We hypothesize that the observed difference in compressive strength of PRM between EICP and MICP treatments is due to the increased deposition of CaCO_3_ with MICP treatment (Figure 2). Hao et al. found that a higher mass of CaCO_3_ coating on plastic increased the fiber pullout strength of plastic from cement mortar up to a peak value, after which pullout strength rapidly decreased [18]. Another potential mechanism is that the increased texture of MICP CaCO_3_ could provide more surface area for interaction with the cement matrix during cement hydration and mechanical loading. Further work is required to explore these hypotheses.

### 3.2. Comparison of MICP Treatment of Type 3–7 Plastics

The results of the EICP and MICP comparison (Section 3.1) were used to inform mineralization strategies for type 3–7 plastics. MICP was chosen to mineralize type 3–7 plastics as it resulted in higher strength PRMs than EICP treatment in comparisons of PET. We chose 5% plastic replacement of cement for type 3–7 plastics based on the results of our PET study. While a modest strength decrease is seen with 5% plastic replacement, it is much lower with MICP treatment compared with untreated plastic and allows for the use of more plastic waste.

**Figure 7:**
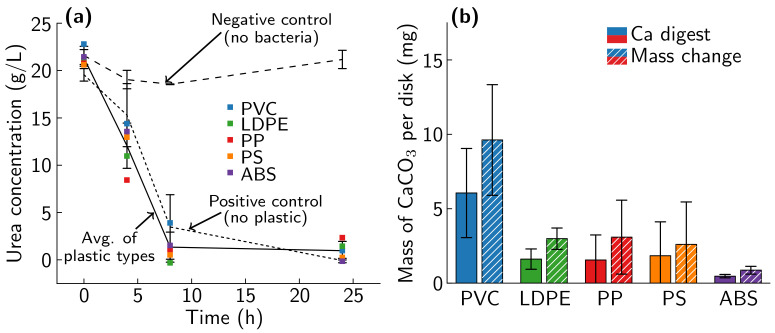
(**a**) Urea concentration results for each plastic type batch test, a no-bacteria negative control, and a plastic-free positive control. (**b**) Mean amount of CaCO_3_ present for each plastic type as measured by mass change (dashed) and calcium assay (solid). Error bars indicate one standard deviation.

**Figure 8:**
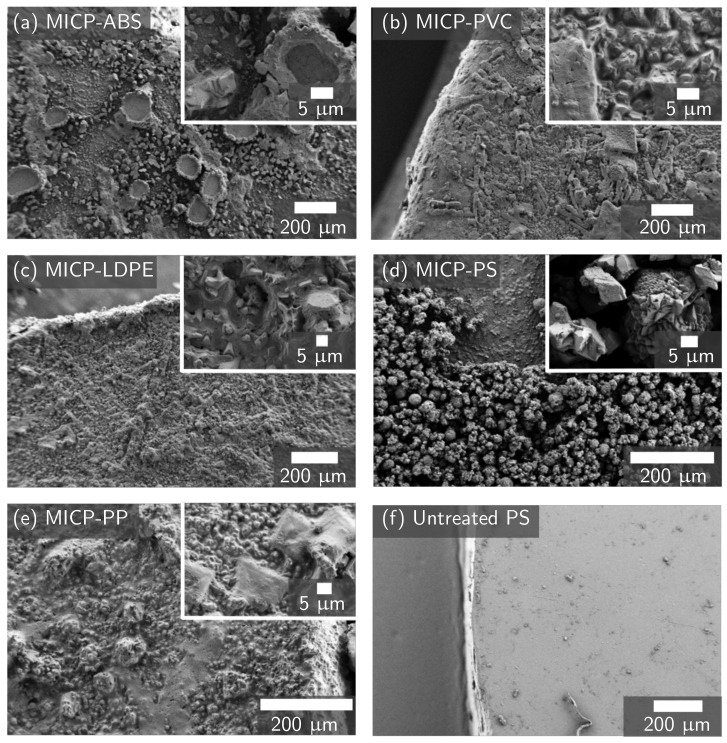
FESEM images of MICP-produced calcium carbonate crust on the surface of (**a**) ABS, (**b**) PVC, (**c**) LDPE, (**d**) PS, and (**e**) PP plastic disks at approximately 300× magnification, with insets at approximately 2500× magnification. Calcium assay results (Figure 7b) show that ABS had the least CaCO_3_ deposition while PVC had the most. (**f**) An untreated PS plastic disk at approximately 300× magnification shows little surface roughness. All other untreated plastics show a similarly smooth surface to PS.

All plastic types 3–7 were successfully biomineralized. OD${}_{600}$, pH (Figure S5), and urea concentration measurements (Figure 7a) all show the biomineralization reaction progressing to the same degree as the plastic-free positive control. However, the amount of CaCO_3_ deposited on the plastic varies by plastic type (Figure 7b). MICP-treatment deposited the least amount of CaCO_3_ on ABS when measured with both mass change and calcium assay, while PVC sees the highest amount of CaCO_3_ deposition with both measurements. PP, PS, and PVC have large variations in the mass of CaCO_3_ deposited, indicating that the mineralization of these samples may not be uniform. Representative FESEM images for each type of plastic show that different MICP-treated plastics have different mineral morphologies (Figure 8). FESEM images of MICP-treated PVC show a uniform mineral coating, with a consistent mineral structure. MICP-treated ABS has more scattered and clumped mineralization. MICP-treated LDPE, PP, and PS each show a unique mineral structure, with LDPE and PP showing consistent mineral structures, similar to PVC, while PS shows a scattered mineral coating, more similar to ABS. The crystal morphology also differs by plastic type, with ABS and PS showing sharp crystals approximately 5 μm in size, while PVC, LDPE, and PP all show smaller, more rounded crystals.

**Figure 9:**
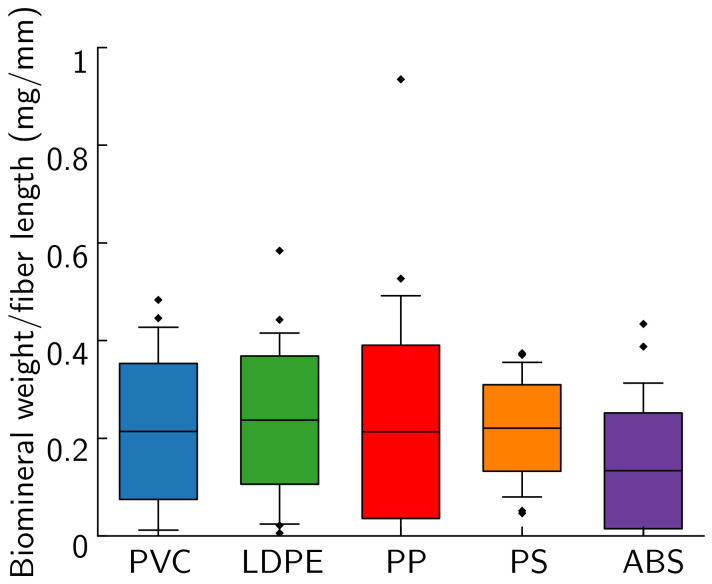
Weight of CaCO_3_ per fiber length on fibers mineralized with MICP via the submersion method. The center line is the median, the colored boxes are the middle 50% of data points, the whiskers contain 95% of data points and the ♦ are outliers.

For the submersion method, on average, 0.22 ± 0.01 mg CaCO_3_ was deposited per mm of fiber length for all plastic types except ABS (Figure 9). ABS had an average of 0.13 mg CaCO_3_/mm of fiber length. Additionally, 14% of ABS fibers measured were found to have no mineral, compared to 6% for PP, 4% for PVC, and 0% for all other plastic types. Additional evidence from the batch tests (Figure 7) and FESEM imaging (Figure 8) leads us to hypothesize that MICP may be less effective at mineralizing ABS than other plastic types. However, additional work is needed to further support this hypothesis and to understand the mechanisms behind the observed differences. Less variation in CaCO_3_ mass between plastic types is observed in these samples than in batch tests (Figure 7a). This demonstrates the success of the submersion method in more uniformly coating plastics than the plastic-in-solution method used in the batch tests.

The amount of CaCO_3_ deposited on plastic with the submersion method was found to be greater than that observed in past studies of plastic treatment with MICP. Hao et al. reported that mineralization of PP with MICP formed 0.026 g CaCO_3_ per g PP after 24 h of mineralization and increasing amounts at longer mineralization times [18]. Our MICP-treated PP shows an average value of 0.098 g CaCO_3_/g PP after 24 h of mineralization. Similarly, PVC (0.064 g CaCO_3_/g PVC), LDPE (0.105 g CaCO_3_ /g LDPE), PS (0.086 g CaCO_3_ /g PS), and ABS (0.052 g CaCO_3_ /g ABS) show more CaCO_3_ deposition then the method used by Hao et al. deposited in 24 h for PP. The mass of CaCO_3_ deposited by the submersion method is similar to the 0.096 g CaCO_3_/g plastic that Hao et al. found to provide the largest increase in fiber pullout strength from cement mortar [18].

**Figure 10:**
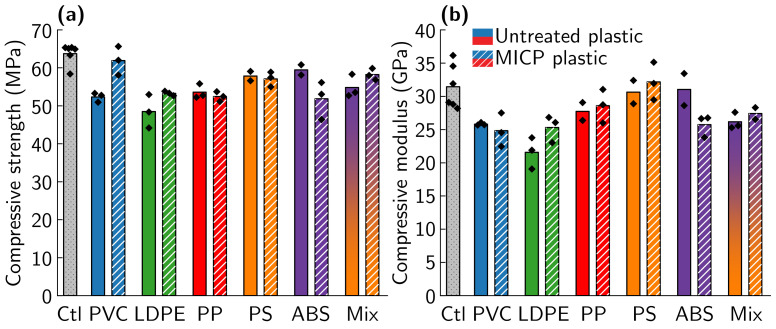
Comparison of compressive strength (**a**) and modulus (**b**) for non-reinforced mortar (Ctl) and MICP-treated and untreated PVC, LDPE, PP, PS, ABS, and mixed-type plastic reinforced mortar. Bars represent mean values and ♦ are individual data points. Exact compressive strength and modulus values are shown in Table S2, and representative stress–strain curves are shown in Figure S7.

MICP treatment increased compressive strength for some, but not all, plastic types (Figure 10a). An interaction between MICP treatment and plastic type was observed for compressive strength of PRM reinforced with untreated and MICP-treated type 3–7 plastics. Post hoc testing shows a significant increase in compressive strength for mortar samples reinforced with MICP-treated PVC relative to mortar samples reinforced with untreated PVC (18% increase, *p* < 0.05). LDPE, PP, PS, ABS, and mixed plastic all show no statistically significant change in PRM for samples treated with MICP relative to untreated plastic of the same type (p>0.05 for all). Mortar reinforced with 5% MICP-treated PVC shows promising strength, with an average compressive strength at 97% of the average of the plastic-free control mortars. Mortar reinforced with 5% mixed type 3–7 plastics also shows high compressive strength, at 91% of the average of the non-reinforced mortar controls. As mixed plastic waste has the advantage of avoiding costly plastic waste sorting, this is an especially promising result. Only untreated LDPE samples show a significant change in compressive modulus relative to the plastic-free controls (p<0.05), indicating that neither the addition of most types of plastic nor mineralization has a significant impact on compressive modulus (Figure 10b). Similar trends are seen for compressive strength and modulus measurements taken after 14 days (Table S2).

**Figure 11:**
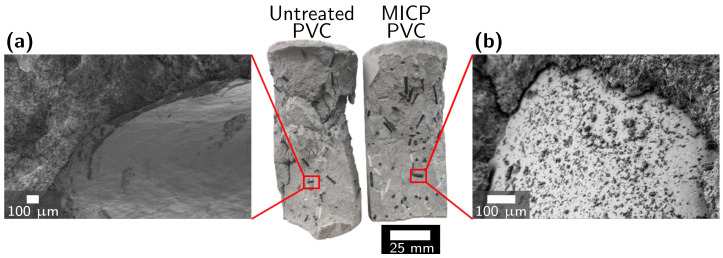
Photos of failed mortar cylinders and FESEM images of the failure surface of cement mortar cylinders reinforced with (**a**) untreated PVC and (**b**) MICP-treated PVC. The interface between plastic and cement is shown. PVC, LDPE, PS, and ABS failure interfaces are shown in Figure S8.

FESEM imaging of the interface between plastic and cement at the fracture surface show evidence supporting a reduction in the gap at the interface between plastic and cement with mineralization (Figure 11 and Figure S8). In images of one sample of PVC, which sees the largest increase in compressive strength with MICP treatment, MICP-treated PVC was observed to have a cement matrix on the surface of the plastic after failure, while untreated PVC does not (Figure 11). This may indicate improved bonding between PVC and the cement matrix. To better understand the failure mechanisms of mineralized PRM and to explain the differences observed between plastic types, additional work is needed to quantify the fiber pullout strength of each plastic type with and without mineralization.

The compressive strengths measured in this study demonstrate that, for PET and PVC plastic, MICP treatment can produce a significant increase in PRM compressive strength while, in other plastic types, little change in compressive strength was seen with mineralization. MICP-treated PP PRM had a statistically insignificant change in compressive strength compared to untreated PP, a finding consistent with the compressive strength testing performed by Hao et al. [18]. It is not yet fully understood why these differences exist between plastic types. The differences in strength between plastic types do not appear to only be related to a change in CaCO_3_ mass and may also be impacted by factors including plastic surface roughness, fiber modulus, or CaCO_3_ surface geometry. Past studies have identified increases in fiber pullout strength, an indicator of improved compressive strength, with MICP treatment of plastic [18] and application of CaCO_3_ to plastic [17]. However, this work is the first to examine the impact of both plastic type and biomineralization on the compressive strength of PRM. Due to MICP previously being used to repair cracks in concrete and seal oil wells [19,23,24,25,26,27], we expect that MICP treatment of plastic has the potential to be scalable for treating plastic waste. An important limitation of the current work is that the influence of each biomineralization method and plastic type on mechanical properties other than compressive strength, such as flexural strength and toughness, workability, and durability, was not examined. Additionally, it would be valuable to examine different plastic geometries and higher plastic volumes to determine optimal geometries for MICP-treated PRM and to maximize the amount of plastic waste used. Importantly, increases in strength over what was observed in this study would be expected if MICP-treated plastics were applied in plastic-reinforced concrete rather than mortar. Based on the compressive strengths for PRM with MICP treatment of PVC, PET, and mixed-type plastic observed in this study, we would expect plastic-reinforced concrete with these treated plastics to have sufficiently high strength for important construction applications, such as concrete slabs, footpaths, and walls [49].

### 3.3. Effect of Biomineral on Cement Hydration

**Figure 12:**
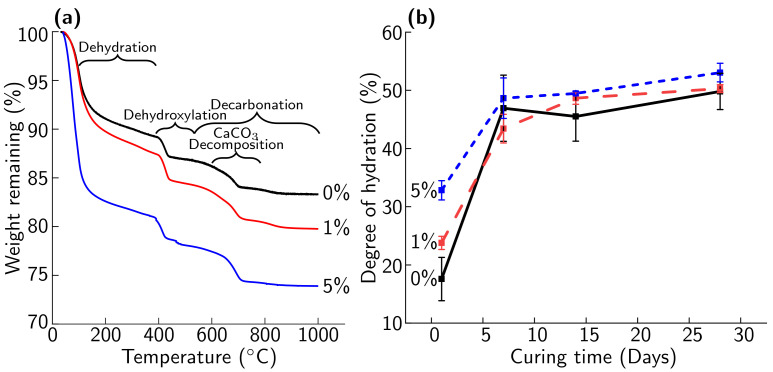
(**a**) Thermogravimetric analysis (TGA) curve of 0% (black), 1% (red), and 5% (blue) biomineral cement paste samples after 1 day of curing; 7, 14, and 28 day and replicate TGA curves are shown in (Figure S9). (**b**) Degree of hydration of 0% (black), 1% (red), and 5% (blue) biomineral cement paste over time, as determined from TGA measurements.

Cement hydration plays an important role in the mechanical properties of cement mortar and concrete. Changes in cement hydration may play a role in the increases in compressive strength with MICP treatment observed in this study [17]. After 1 day of curing, cement hydration, as measured via TGA, increases with the amount of biomineral added (Figure 12b). This increase in measured rate of hydration is consistent with the formation of a denser cement matrix at the interface around biomineralized fibers that has been observed in past studies of CaCO_3_-treated PRM [17]. The largest difference in cement hydration was found at a curing time of 1 day, with small increases in hydration observed at 7, 14, and 28 days. The increase in hydration with biomineral after 1 day of curing was observed as being due to an increase in mass loss during the dehydration, dehydroxylation, and decarbonation degradation regions (Figure 12a). At longer curing times, similar amounts of mass loss are seen in the decarbonation and dehydration regions for 0%, 1%, and 5% biomineral samples.

The 5% biomineral sample experienced a larger mass loss than 0% and 1% biomineral samples in the dehydroxylation region and a larger mass loss in the decomposition of the CaCO_3_ region (Figure 12a) [37]. This contribution to the mass loss indicates that, in the 5% biomineral cement paste samples, more portlandite was formed than at 1% and 0% biomineral. Calcium present in the CaCO_3_ biomineral can participate in the cement hydration reaction [50]. The presence of an additional mass loss in the decomposition of the CaCO_3_ region indicates that a portion of the biomineral has not participated in the hydration reaction and that unreacted CaCO_3_ remains [50]. We hypothesize that, as biomineral concentrations will be greater than 5% in the regions immediately surrounding biomineralized plastic in the mortar, some inert biomineral will remain surrounding the plastic and a similar effect will be seen on the cement matrix of biomineralized PRM as in the 5% biomineral cement paste sample.

**Figure 13:**
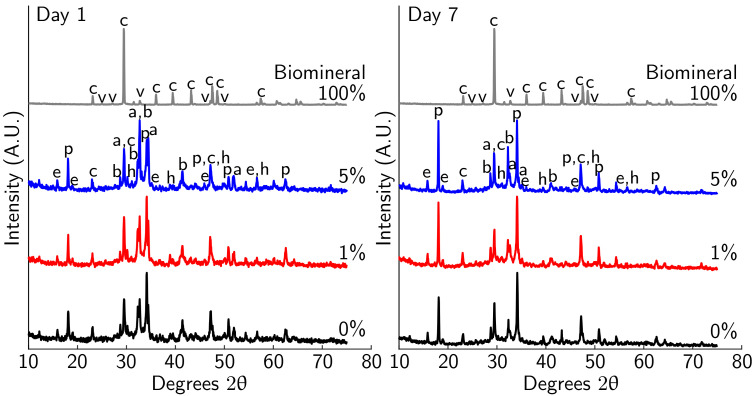
XRD spectrum for cement paste after 1 and 7 days of curing with 0%, 1%, and 5% MICP biomineral added and 100% MICP biomineral. The peaks present are alite (a), belite (b), calcite (c), portlandite (p), ettringite (e), and hemicarboaluminate (h), with labels corresponding to the first letter of the mineral. Little change was seen after 7 days, and XRD spectra for 14 and 28 days are included in the Supplementary Materials (Figure S10).

The XRD results show only minor differences between cement paste samples with 0%, 1%, and 5% biomineral after 7 days of curing (Figure 13). As in TGA measurements, the largest change is observed between 1 and 7 days, with only small differences over time after 7 days of curing (hydration at 1 and 7 days shown in Figure 13 and at 14 and 28 days in Figure S10). Calcite, portlandite, and alite peaks are observed as major phases for all cement paste samples. Ettringite and belite shift between major and minor phases, with the relative phase composition changing with hydration between time points. The monocarboaluminate and hemicarboaluminate phases are observed in small quantities. The XRD spectrum of the biomineral shows a majority calcite structure, with a minor vaterite phase, confirming the results for MICP mineral seen on PET flakes (Figure 2a). Past work by Monteiro et al. on abiotic vaterite-containing carbonate precipitates found that the addition of both calcite and vaterite to cement paste leads to the formation of monocarboalluminate and hemicarboaluminate phases [39]. The XRD results further support an incomplete reaction of the CaCO_3_ polymorphs as cement hydration progresses that was observed in TGA measurements, as evidenced by the remaining calcite and vaterite present in the hydrated samples.

## 4. Conclusions

In this study, we show that biomineralization can improve the strength of plastic-reinforced mortar. A key finding of this study was that none of the examined plastics (PET, PVC, LDPE, PP, PS, or ABS) inhibited *S. pasteurii* growth under the biomineralization conditions tested in this study. MICP treatment deposited more CaCO_3_ on the surface of PET plastic than EICP treatment, resulting in PRM with 5% MICP-treated PET having 88% of the compressive strength of the plastic-free mortar. Importantly, PRM reinforced with 5% MICP-treated PET, PVC, and mixed type 3–7 plastics had strengths similar to that of plastic-free mortar and showed sufficient strength for application in engineering structures.

Together, these results indicate that MICP treatment allows for the reuse of larger volumes of plastic waste in PRM. At constant values of plastic addition, MICP also increases the strength of PRM, which may be valuable in applications where high strength is a concern. As mixed type 3–7 plastic is a low-value, often landfilled plastic waste stream, this treatment is a promising option to reduce the amount of plastic waste sent to landfills. Biomineralized mixed-type PRM shows similar strength to non-reinforced mortar, indicating that mixed-plastic waste may be added in many concrete applications to reuse this low value waste stream.

Additional work is needed to better understand why MICP treatment affects the compressive strength of PRM differently in each plastic type. This study observed an increase in cement hydration and a visually improved interface between plastic waste and cement mortar with MICP treatment. To better understand the mechanisms behind the differences in strengths observed in this study, further work is needed to directly establish the impact of MICP treatment on interfacial strength. Increased knowledge of these mechanisms may allow additional improvement in the strength of PRCs with MICP treatment. These improvements would further establish biomineralized PRC as a high-volume method to reuse plastic waste.

## Figures and Tables

**Table 1:** Experimental matrix for both polyethylene terephthalate (PET) and plastic types 3–7 microbially induced calcium carbonate precipitation (MICP) batch study.

	Attachment Phase	Mineralization Phase
Test condition	*S. pasteurii*, plastic, CMM−	Plastic (with biofilm), CMM+
Positive control	*S. pasteurii*, CMM−	None
Negative control	Plastic, CMM−	Plastic (no biofilm), CMM+

**Table 2:** Cement mortar cylinder specimen experimental matrix.

Plastic Type (s)	Mineralization Method	wt.% Replacement
PET	EICP	1%, 5%
PET	MICP	1%, 5%
PET	None	1%, 5%
Types 3–7	MICP	5%
Types 3–7	None	5%
None (control)	None	0%

**Table 3:** Change in mean cement mortar compressive strength relative to the mean for non-reinforced mortar control when reinforced with untreated PET, EICP-treated PET, or MICP-treated PET at 1% or 5% replacement after 35 days of curing.

	Change in Compressive Strength Relative to Control		
**Plastic Replacement**	**Untreated PET**	**EICP-PET**	**MICP-PET**
1%	−14.6%	−3.74%	−2.54%
5%	−30.1%	−29.2%	−11.9%

## Data Availability

The data presented in this study are openly available in Dryad at https://doi.org/10.5061/dryad.79cnp5hvg.

## Supplementary Materials

The following are available at https://www.mdpi.com/article/10.3390/ma14081949/s1, Table S1: Plastic properties, Figure S1: Submersion method setup, Figure S2: Plastic fiber length, Figure S3: PET additional batch test data, Figure S4: Untreated PET FESEM image, Figure S5: Type 3–7 additional batch test data, Table S2: Full PRM strength data, Figure S6: PET PRM stress-strain curves, Figure S7: Type 3–7 PRM stress-strain curves, Figure S8: Type 3–7 PRM interface FESEM images, Figure S9: Cement hydration TGA curves, and Figure S10: Cement hydration XRD spectra.
