# Supplementary material for: Biomineralization of Plastic Waste to Improve the Strength of Plastic-Reinforced Cement Mortar
Source: Materials (Basel). 2021 Apr 13;14(8):1949. doi: 10.3390/ma14081949 (PMC8069578; doi:10.3390/ma14081949)
Supplement: Supplementary file 1 [file materials-14-01949-s001.pdf]

Supplementary Materials: Biomineralization of Plastic Waste to Improve the Strength of Plastic-Reinforced Cement Mortar

Seth Kane[1,2], Abby Thane[2], Michael Espinal[1,2], Kendra Lunday[4], Hakan Armağan[5], Adrienne Phillips[3,2], Chelsea Heveran[1,2], and Cecily Ryan[1,2]

The figures and text presented here were prepared in support of the methods and results of "Biomineralization of plastic waste to improve the strength of plastic-reinforced cement mortar". Section 1 provides additional methods, supporting the setup of the confocal images taken. Figure S1 provides additional detail on the dunk tank method experimental setup and Figure S2 shows the distribution of fiber lengths used for type 3–7 plastics.

Section 2 provides additional results, with Figures S3 and S5 provide additional data from the MICP treatment batch test for PET and type 3–7 plastic respectively. Figure S4 shows an SEM image of an untreated PET flake, in support of Figure 3 of the main text. Figures S6 and S7 show representative stress-strain curves for comparisons of EICP and MICP treatment of PET and MICP-treatment of type 3–7 plastic respectively. Table S1 displays compressive strength and modulus values for all PRM samples measured in this study at both 14 and 28 days of curing time. Figure S8 shows SEM images of the interface between the cement matrix and plastic for MICP-ABS, MICP-LDPE, MICP-pp, Untreated PP, MICP-PS, and untreated PS. SEM images of the interface for EICP, MICP and untreated PET and MICP and untreated PVC are available in Figures 5 and 10 of the main text respectively. Figures S9 and S10 show TGA curves and XRD data for cement paste samples with 0%, 1% and 5% MICP biomineral added after 1, 7, 14 and 28 days of curing. For more information, refer to the main text.

1. Supplemental Methods

1.1. Supplemental Confocal Methods

Confocal images were collected through a Leica ELWD 10×/0.3 water objective. The 3D confocal stacks were taken at every 6.42 µm, with a voxel size of 1.52 × 1.52 × 6.43 µm and a total vertical thickness of 192.6 µm. The data sets were imported into IMARIS 9.2 and the individual stacks were merged into a 2D image. The sample was stained with ThermoFisher LIVE/DEAD BacLight Bacterial stain. The fluorescent signal for stained bacterial cells was collected by excitation with Argon laser at 488 nm and the photomultiplier tube detection (PMT) between 550–630 nm (colored green). The natural autofluorescence of calcium carbonate was captured by excitation at 405 nm and PMT detection between 450–500 nm (shown in red). The overlay of red and green, where both the mineral and cells are present is represented by yellow.

1.2. Tensile Measurement of Plastic Fibers

Plastic fiber mechanical properties were determined by tensile testing on an Instron 5543 Universal Testing System, with a loading rate of 5 mm/min. and the distance between the clamps was 30 mm. Samples were mesaured in triplicate.

Table S1. Hot pressing temperature, density, tensile strength, and elastic modulus for all plastic filament examined in this study. Densities are provided by the manufacturers and tensile strength and elastic modulus were calculated as described in Supplemental Section 1.2.

	PVC	LDPE	PP	PS	ABS
Hot pressing temperature (°C)	180	105 C	18	220	220
Density (g/cm^3)	1.35	0.92	0.75	1.03	1.04
Tensile Strength (MPa)	45.5	9.89	11.61	18.2	44.31
Elastic Modulus (GPa)	1.292	0.16	0.3	0.65	0.91

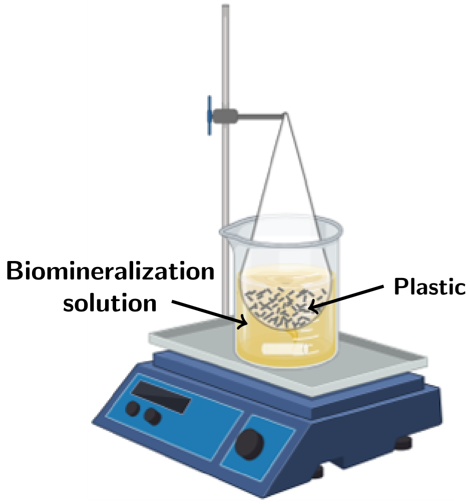

Figure S1. Diagram of the setup of the dunk tank reactor used for MICP treatment of PVC, LDPE, PP, PS and ABS plastic fibers. The dunk tank setup allows for fibers with a density lower than water to remain fully submerged throughout the mineralization process.

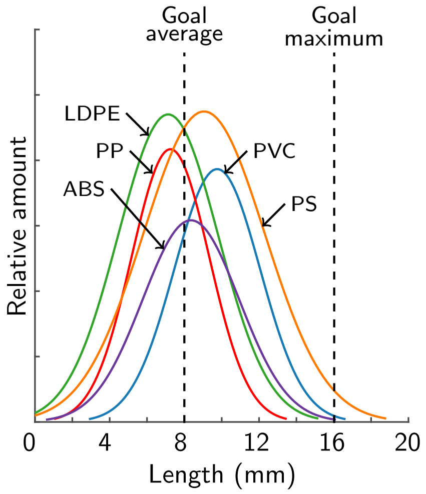

Figure S2. Distribution of fiber lengths for all plastic types. All fiber types had a goal length of 8 mm. A goal maximum length of 16 mm was established based on ASTM C1609 [1].

2. Supplemental Results

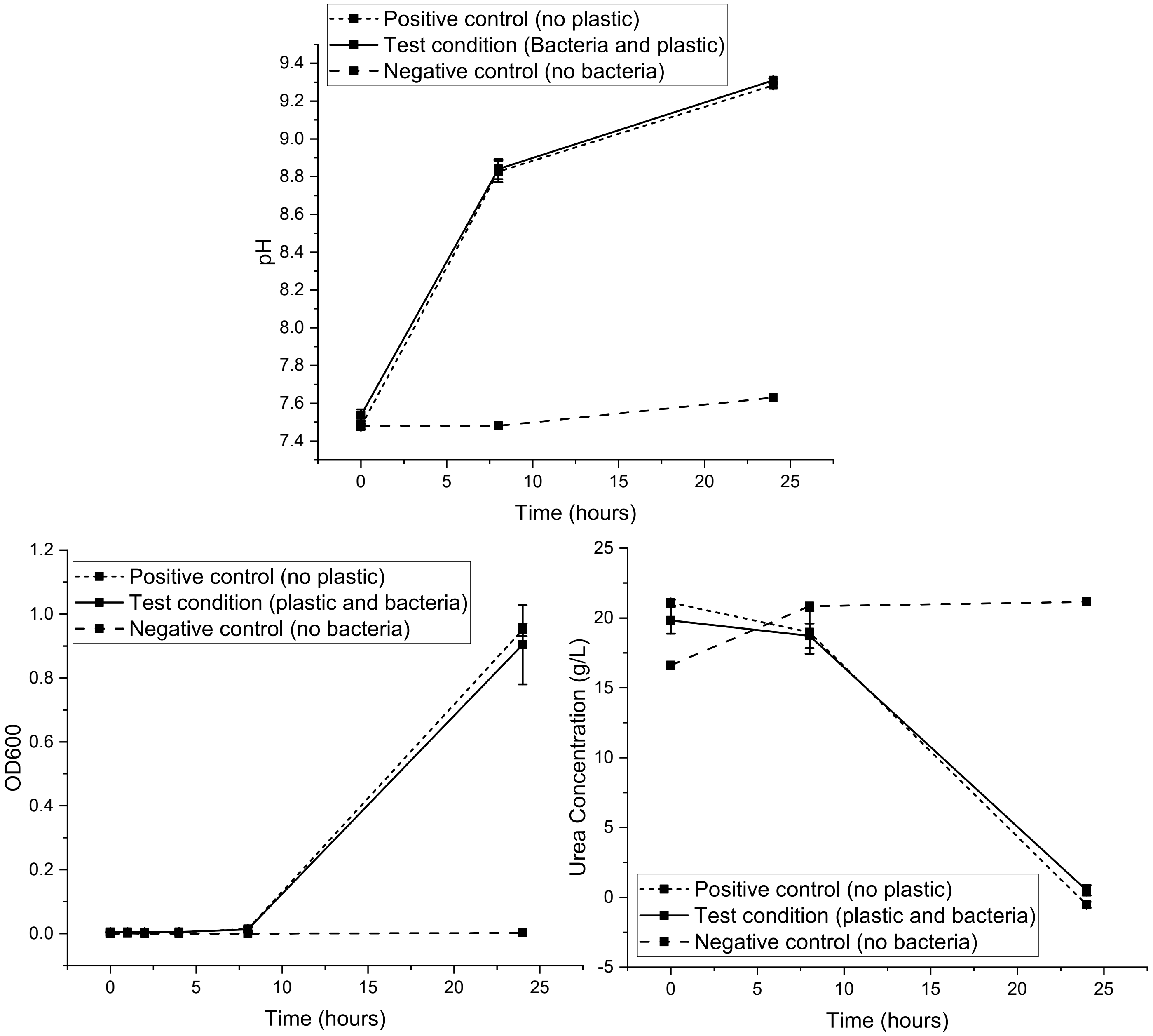

Figure S3. pH, OD600 and urea concentration data from the batch test of MICP treatment of PET plastic. The data shows mineralization of samples with PET progressing at the same rate as samples without PET. Error bars represent one standard deviation.

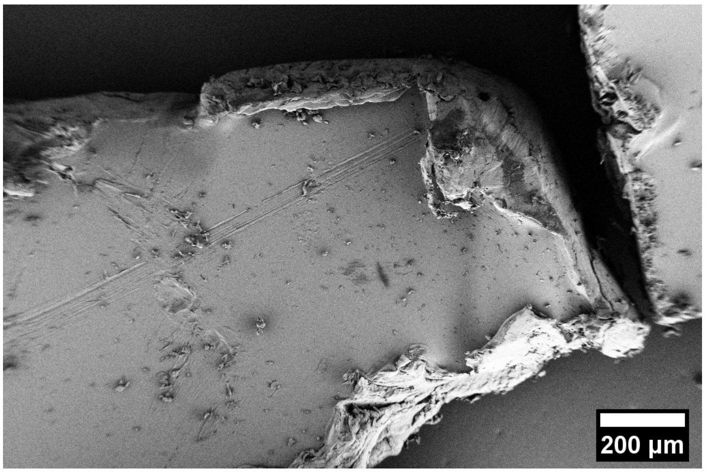

Figure S4. An SEM image of an untreated PET flake at approximately 200× magnification.

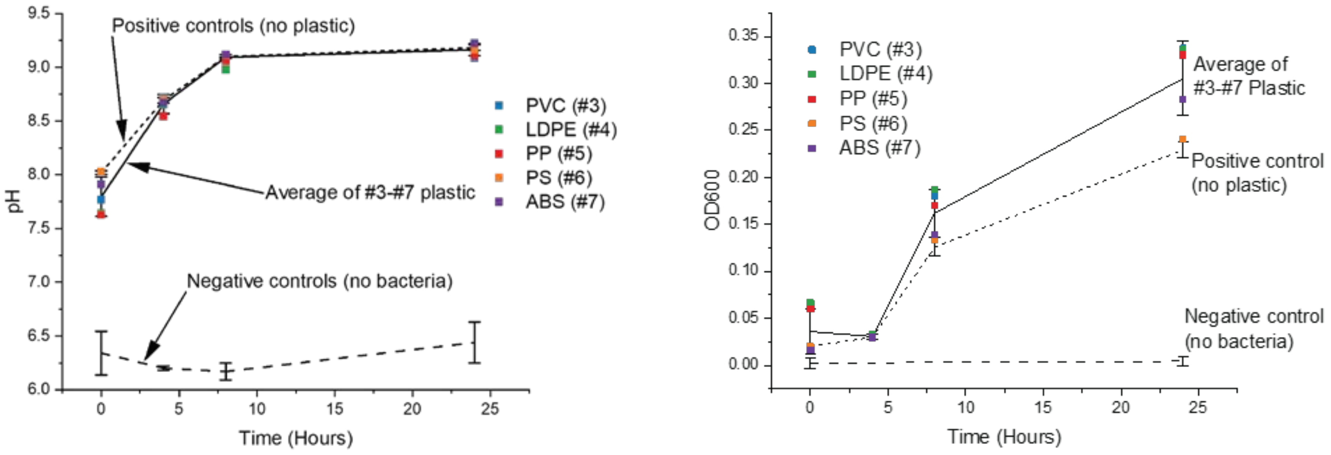

Figure S5. pH and OD600 data from the batch test of MICP treatment of type 3–7 plastic. Urea concentration is shown in Figure 6 of the main text. Error bars represent one standard deviation.

Table S2. Means and standard deviations of compressive strength and modulus for all PRM samples measured in both studies comparing EICP and MICP treatment and MICP treatment of type 3–7 plastic. For the plastic type column, the amount of plastic is included followed by the plastic type (1 PET, 3 PVC, 4 LDPE, 5 PP, 6 PS, 7 ABS, and Mix as a mixture of type 3–7 followed by the mineralization treatment, where u = untreated, m = MICP, and e = EICP.

	Plastic Type	14 Days Curing Compressive Modulus (GPa)	14 Days Curing Compressive Strength (MPa)	28 Days Curing Compressive Modulus (GPa)	28 Days Curing Compressive Strength (MPa)
Type 3–7 Plastic Comparison	None	25.550 ± 3.463	49.304 ± 3.733	30.750 ± 2.955	63.745 ± 2.719
	5% 3u	25.179 ± 9.584	44.519 ± 1.010	24.925 ± 1.830	52.326 ± 1.214
	5% 3m	21.057 ± 4.100	44.112 ± 10.806	26.246 ± 2.376	61.899 ± 3.783
	5% 4u	23.979 ± 2.188	38.007 ± 2.078	16.616 ± 3.057	48.507 ± 4.411
	5% 4m	23.039 ± 3.588	42.978 ± 4.275	23.081 ± 3.165	53.274 ± 0.592
	5% 5u	21.581 ± 6.801	44.022 ± 5.583	27.187 ± 2.198	53.595 ± 1.925
	5% 5m	24.994 ± 1.070	44.796 ± 1.393	27.754 ± 1.752	52.437 ± 1.292
	5% 6u	27.556 ± 1.228	47.586 ± 0.207	26.198 ± 5.895	52.774 ± 8.839
	5% 6m	27.195 ± 2.290	49.211 ± 0.681	27.279 ± 4.935	57.099 ± 1.945
	5% 7u	25.548 ± 1.528	47.962 ± 2.895	25.421 ± 8.848	59.466 ± 1.911
	5% 7m	22.409 ± 3.179	42.842 ± 2.101	25.601 ± 1.160	51.854 ± 4.979
	5% Mix u	21.314 ± 2.972	44.459 ± 1.837	22.413 ± 1.227	54.844 ± 3.020
	5% Mix m	25.546 ± 2.039	47.469 ± 2.068	26.698 ± 1.460	58.231 ± 1.523
EICP & MICP	None†	21.521 ± 1.519	49.080 ± 2.657	23.618 ± 0.959	60.769 ± 2.187
	1% 1u†	19.955 ± 1.685	54.028 ± 0.985	22.015 ± 0.459	52.080 ± 4.786
	1% 1e†	19.810 ± 1.577	38.468 ± 1.158	20.538 ± 2.352	42.623 ± 1.540
	1% 1m†	22.577 ± 0.393	47.860 ± 1.743	25.011 ± 2.197	58.536 ± 5.061
	5% 1u†	18.065 ± 2.370	40.924 ± 2.002	19.603 ± 0.228	43.013 ± 5.067
	5% 1e†	19.469 ± 0.922	49.921 ± 1.748	19.600 ± 1.304	59.285 ± 3.125
	5% 1m†	22.304 ± 1.487	49.246 ± 0.890	25.799 ± 0.563	53.561 ± 1.473

† EICP and MICP comparison second measurements were performed at 35 days rather than 28 days.

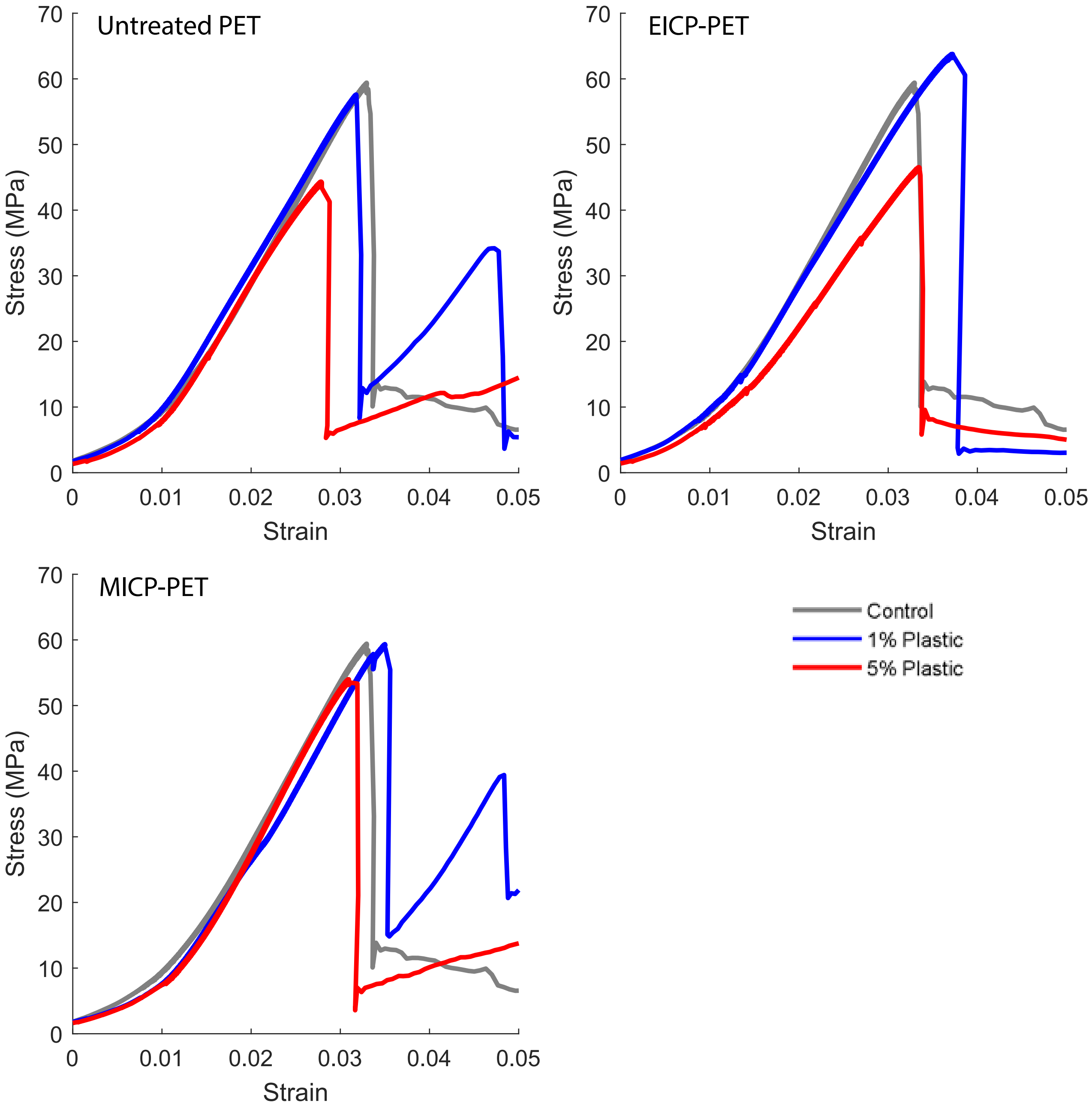

Figure S6. Stress strain curves for PRM reinforced with untreated, EICP-, and MICP-treated PET at 1% and 5% replacement levels. One representative curve for each treatment is shown.

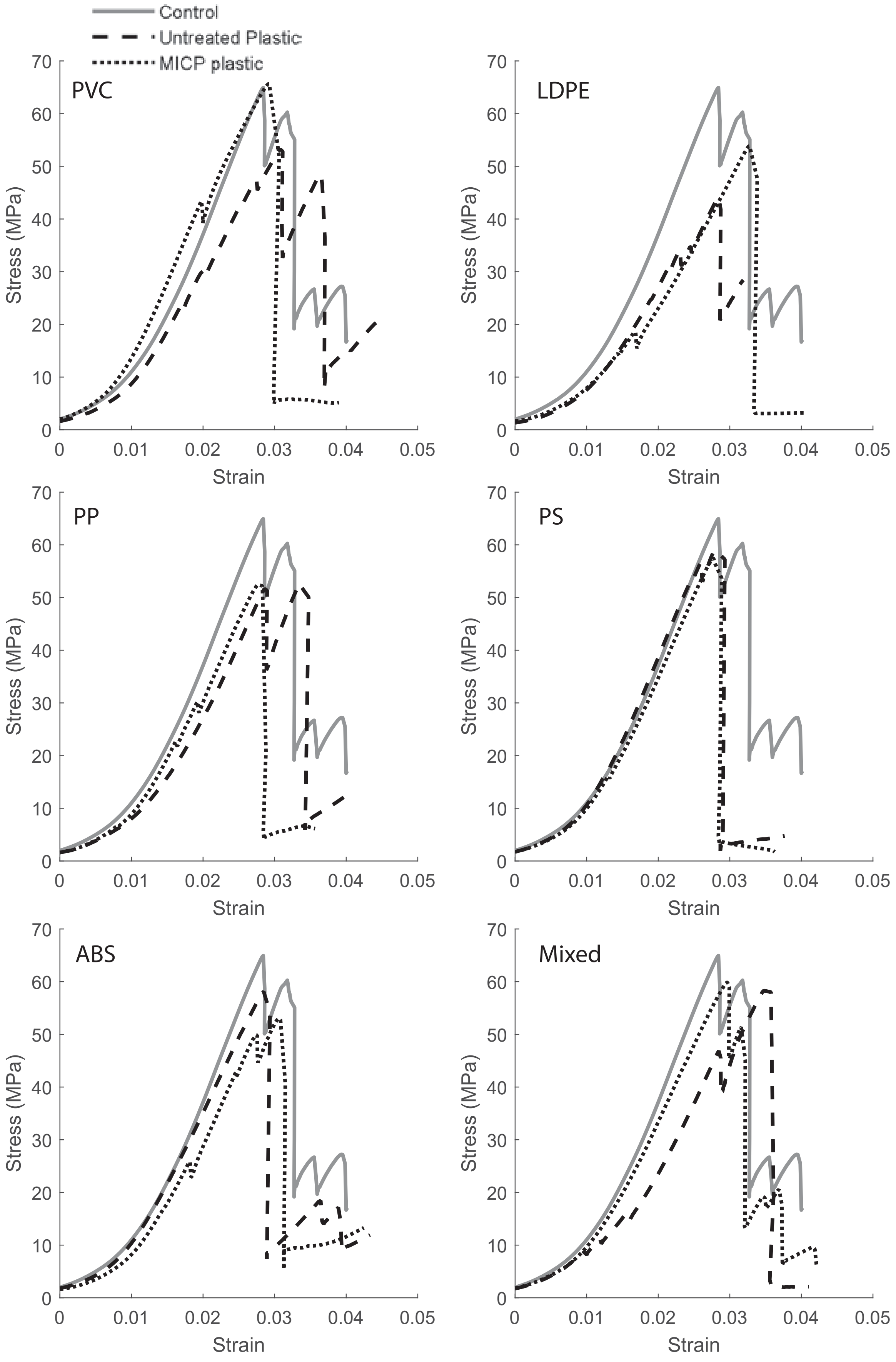

Figure S7. Stress strain curves for PRM reinforced with untreated and MICP-treated PVC, LDPE, PP, PS, ABS and mixed plastic fibers. One representative curve for each treatment is shown.

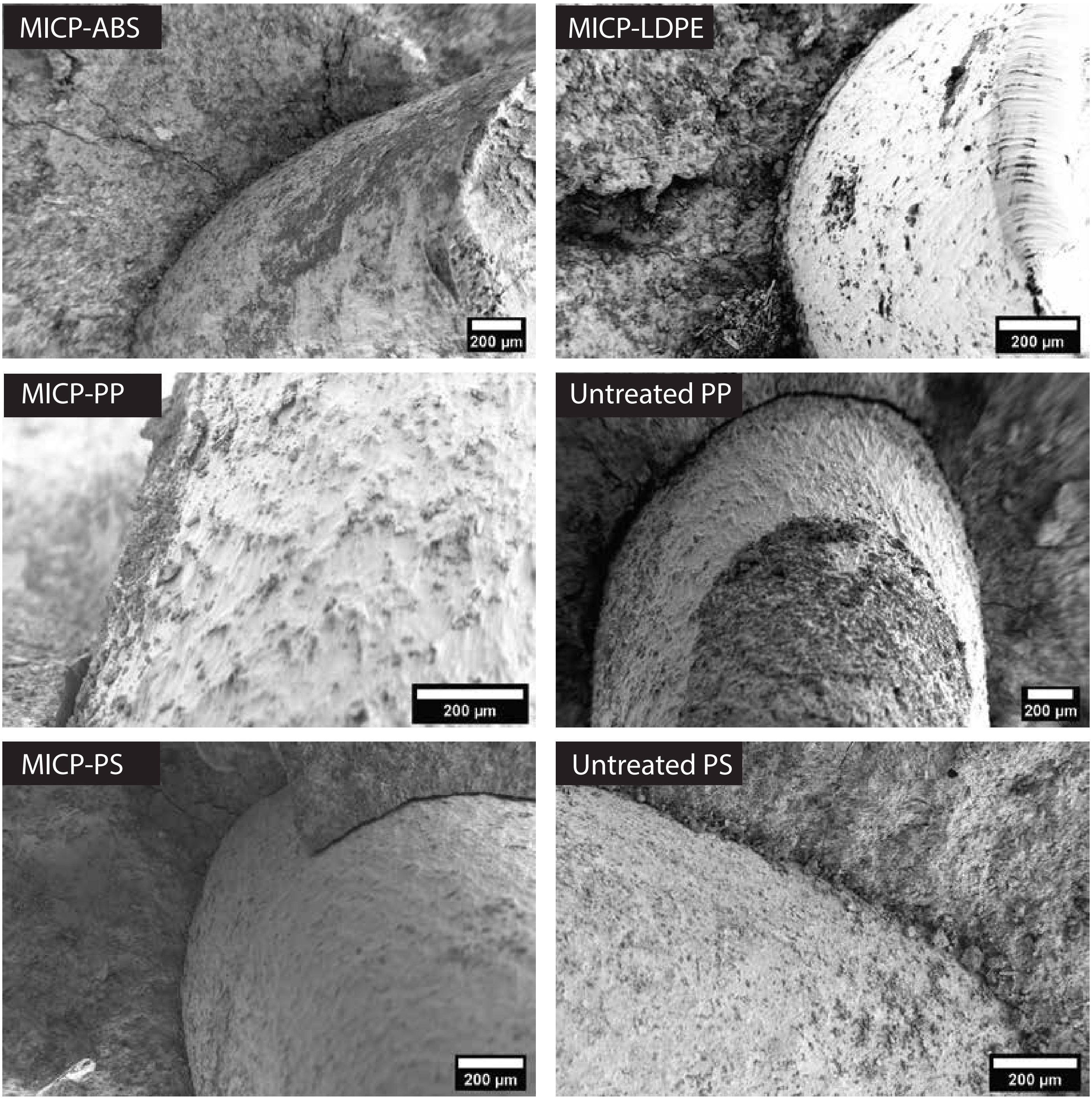

Figure S8. FESEM images of the interface between cement matrix and plastic for MICP-ABS, MICP-LDPE, MICP-PP, untreated PP, MICP-PS and untreated PS at approximately 100× magnification.

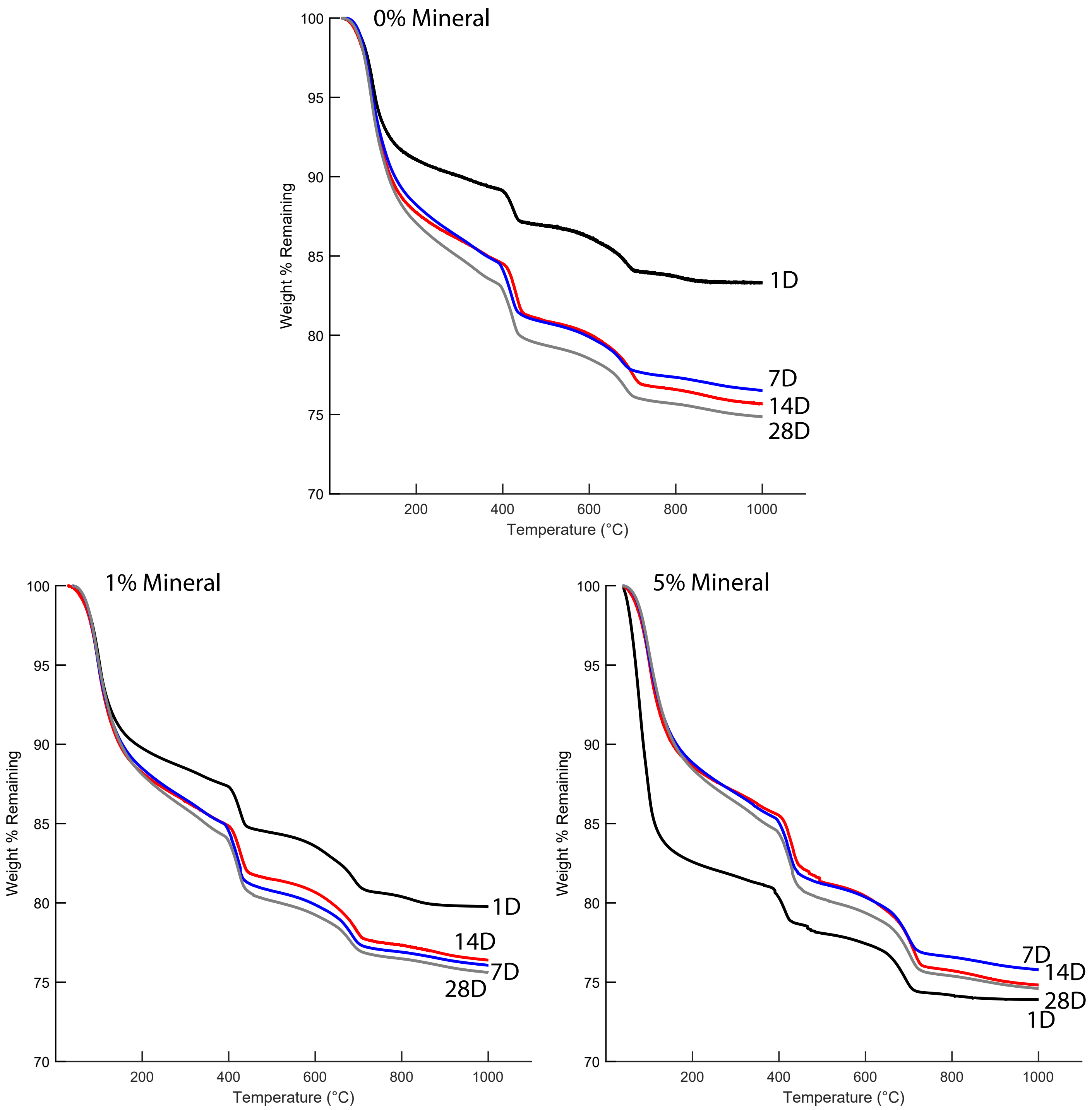

Figure S9. TGA curves used to determine hydration for cement paste with 0%, 1% and 5% biomineral added after 1, 7, 14 and 28 days of curing.

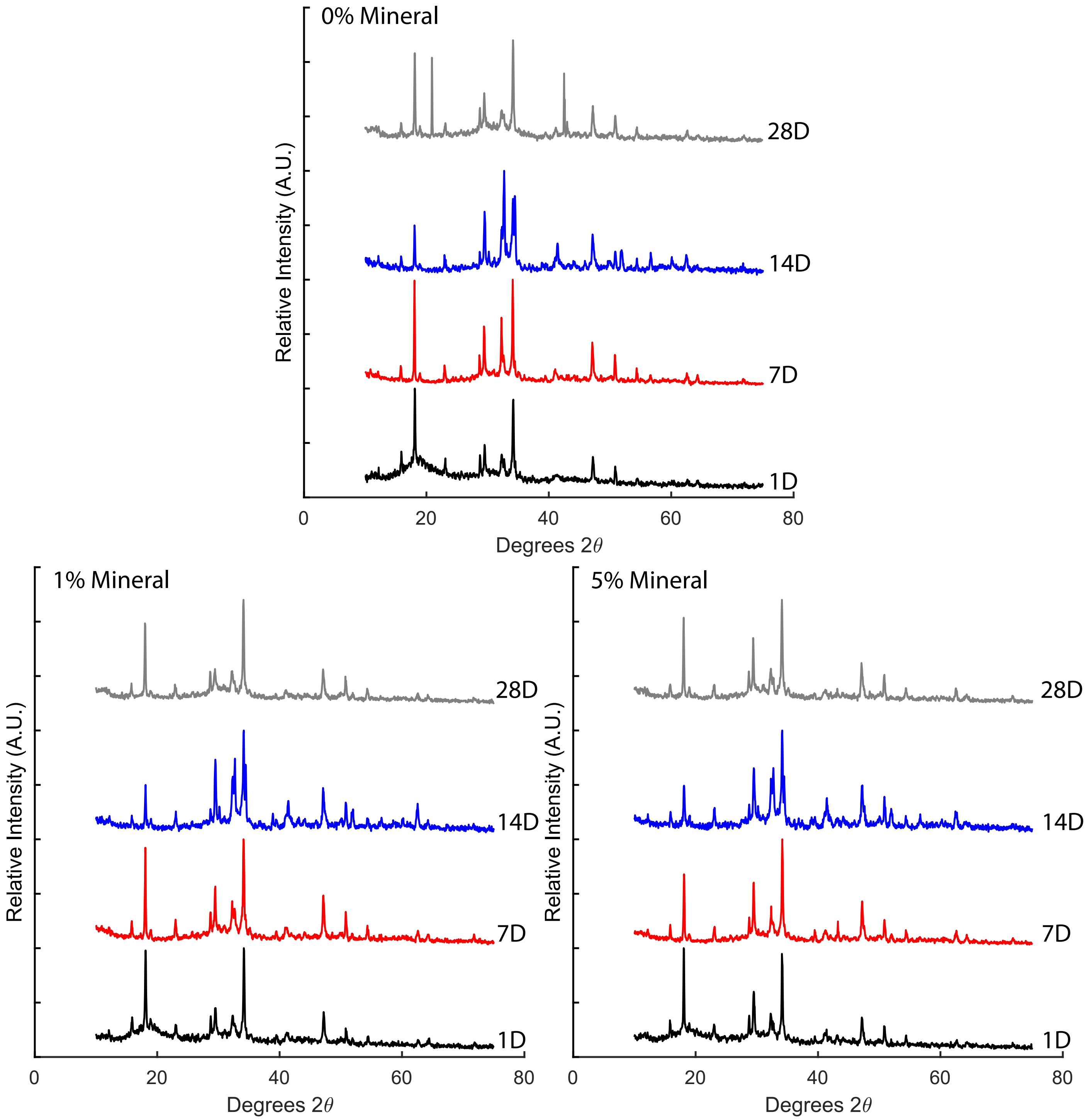

Figure S10. XRD spectra for cement paste with 0%, 1% and 5% biomineral added after 1, 7, 14 and 28 days of curing. See the main text for peak assignments.

Reference

1. ASTM International. C1609/C1609M-19a Standard Test Method for Flexural Performance of Fiber-Reinforced Concrete (Using Beam With Third-Point Loading). Standard, 2019. doi:10.1520/C1609_C1609M-19A.
